# Pyramiding of Multiple Genes to Improve Rice Blast Resistance of Photo-Thermo Sensitive Male Sterile Line, without Yield Penalty in Hybrid Rice Production

**DOI:** 10.3390/plants12061389

**Published:** 2023-03-21

**Authors:** Pei Peng, Haoyu Jiang, Lihua Luo, Changrong Ye, Yinghui Xiao

**Affiliations:** 1 College of Agronomy, Hunan Agricultural University, Changsha 410128, China; 2 Huazhi Biotech Co., Ltd., Changsha 410125, China

**Keywords:** hybrid rice, blast resistance, marker-assisted selection, thermo-sensitive genic male sterile line, gene pyramiding

## Abstract

Rice blast caused by pathogenic fungus *Magnaporthe oryzae* is one of the most serious diseases in rice. The pyramiding of effective resistance genes into rice varieties is a potential approach to reduce the damage of blast disease. In this study, combinations of three resistance genes, *Pigm*, *Pi48* and *Pi49*, were introduced into a thermo-sensitive genic male sterile (PTGMS) line Chuang5S through marker-assisted selection. The results showed that the blast resistance of improved lines increased significantly compared with Chuang5S, and the three gene pyramiding lines (*Pigm + Pi48 + Pi49*) had higher rice blast resistance level than monogenic line and digenic lines (*Pigm +Pi48*, *Pigm + Pi49*). The genetic backgrounds of the improved lines were highly similar (>90%) to the recurrent parent Chuang5S by using the RICE10K SNP chip. In addition, agronomic traits evaluation also showed pyramiding lines with two or three genes similar to Chuang5S. The yields of the hybrids developed from improved PTGMS lines and Chuang5S are not significantly different. The newly developed PTGMS lines can be practically used for the breeding of parental lines and hybrid varieties with broad spectrum blast resistance.

## 1. Introduction

Rice is one of the most important crops in the world, feeding more than half of the world’s population. More than 60% of the population in China take rice as their staple food [1]. Rice blast, caused by pathogenic fungus *Magnaporthe oryzae*, is one of the most serious diseases affecting rice production [2]. The use of a resistance gene to create new cultivars with broad-spectrum disease resistance has been considered a cost-effective and environmentally friendly method to control the disease.

So far, more than 100 resistance loci have been identified, and among them, 38 R genes have been successfully cloned [3,4]. Most of the genes are distributed in the form of gene clusters on the other 11 chromosomes except the third chromosome. The *Piz* locus on chromosome 6, the *Pik* locus on chromosome 11 and the *Pita* locus on chromosome 12 are the hotspots of resistance gene clusters [5]. Most importantly, R genes in these hotspot regions often show broad-spectrum resistance to rice blast fungus, which provides important genetic resources for breeding rice varieties with broad-spectrum blast resistance [6].

The broad-spectrum rice blast resistance gene *Pigm* at the *Piz* locus has both leaf blast and panicle blast resistance effects and has been widely used in rice breeding [7,8]. Gene *Pi49* is a new broad-spectrum resistance gene at the *Pik* locus and discovered in the landrace Mowanggu, which has also been used in rice blast resistance breeding in recent years [9]. Genes *Pi47* and *Pi48* were identified in the *indica* cultivar Xiangzi 3150 and located at the *Pik* and *Pita* locus, respectively; they determined the stable broad-spectrum resistance of Xiangzi 3150, which conferred resistance to 95% of 303 blast isolates from China [10].

Chuang5S is an *indica* thermo-sensitive genic male sterile (TGMS) rice line bred by Hunan Agricultural University; it has excellent agronomic characteristics, such as lower critical sterility inducing temperature (refers to the critical temperature when a sterile line changes from a sterile state to a fertile state), stable sterility and a high combining ability and outcrossing rate [11]. More than ten hybrid combinations bred from Chuang5S have been approved and widely cultivated. However, it was found in the production practice that the rice blast resistance of Chuang5S was poor, and there is risk involved by planting the hybrids from this male sterile line in certain areas [12,13]. Therefore, in this study, three blast resistance genes (*Pigm*, *Pi48*, *Pi49*) were introgressed into Chuang5S by combining the traditional breeding method with MAS technology to develop new PTGMS lines with resistance to rice blast, aiming to improve the disease resistance of two-line hybrid rice bred from Chuang5S. Meanwhile, we also evaluated the yield of hybrid combinations derived from improved lines with one, two or three resistance genes in order to explore whether the yield was affected by the number of resistance genes.

## 2. Materials and Methods

### 2.1. Plant Materials

**Figure 1:**
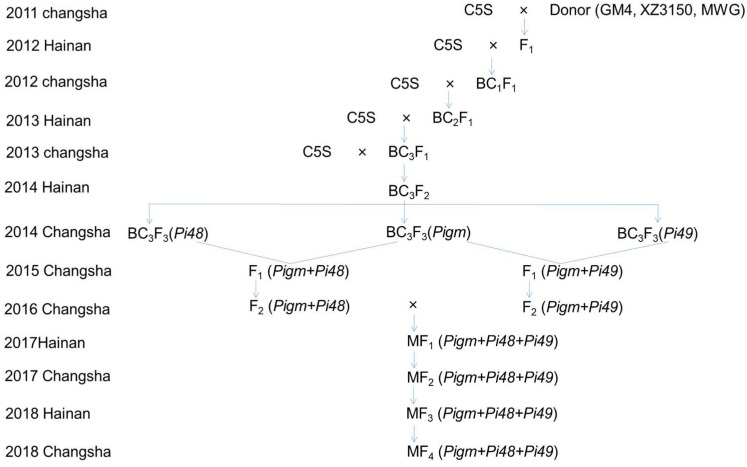
Breeding scheme for developing introgression lines of C5S carrying rice blast resistance genes *Pigm*, *Pi48* and *Pi49*.

In this study, Chuang5S (C5S) and NIL-C5S carrying the R gene were used as the recurrent parents. Blast resistance gene *Pigm* was derived from a native variety Gumei 4 (GM4). Dominant blast resistance gene *Pi48* was derived from Xiangzi 3150 (XZ3150), a local rice variety in Hunan Province of China. Long-lasting blast resistance gene *Pi49* was derived from Mowanggu (MWG), a local variety in Yunnan Province of China. Rice variety CO39 was used as the susceptible control. A cross was made between Chuang5S and donor parents to generate the F_1_ hybrids during the summer of 2011 at Changsha. After three backcross generations, the target BC_3_F_3_ lines were obtained. BC_3_F_3_ plants containing different resistance genes were crossed to generate new F_1_ hybrids, then F_1_ were self-pollinated to obtain the F_2_ population. Then, the F_2_ plants containing different resistance genes were crossed to generate the F_1_ hybrids which harbored multiple resistance genes. After three self-pollination generations, the pedigree selection was followed to obtain the target MF_4_ lines (Figure 1).

### 2.2. Molecular Marker Selection and Genotyping

Molecular markers linked to the target genes were selected based on previous research; a set of molecular markers with polymorphism among parents were selected for foreground selection (Table 1). SSR markers RM7178 and RM7311 were used for *Pigm* detection, LY2 was used to detect *Pi48* and RM224 was used to detect *Pi49* [12,14].

DNA samples were prepared by using the SDS-CTAB method and sucrose extraction method [15]. The PCR system (10 μL) for amplifcation was as follows: 1.0 μL 10 × buffer, 0.2 μL 5-mM dNTPs, 1.0 μL 2 pmol/μL primers, 0.1 μL 5 U/μL Taq polymerase, 1.0 μL DNA template (about 10 ng/μL) and 6.7 μL ddH_2_O. The PCR was performed on the ABI PCR system 2700. The PCR procedure was 94 °C for 5 min, followed by 35 cycles of 94 °C for 30 s, 55 °C for 1 min, 72 °C for 30 s and finally 72 °C for 10 min. The PCR products were separated via electrophoresis on 8% non-denaturing polyacrylamide gel, and then visualized via silver staining and finally observed using a fluorescent light box.

### 2.3. Genetic Background Examination of Pyramided Lines

The whole-genome single-nucleotide polymorphism (SNP) array RICE10K was used to evaluate the similarity in genetic background between the improved lines and the recurrent parent C5S. This SNP array was developed based on a multiplex PCR and genotyping using targeted sequencing technology and comprises 9906 high-quality SNP and insertion-deletion (InDel) markers evenly distributed on the 12 chromosomes of rice with an average density of 27 SNPs per Mb. The pyramided lines of MF_6_, F_8_ and BC_3_F_11_ were selected and genotyped for examination of the genetic background. For each improved line or C5S, the total DNA was extracted from the leaves of 30 plants. Genetic background similarity analysis was performed at China National Hybrid Rice Research and Development Center (Changsha, China) according to Genotyping by Targeted Sequencing Protocol.

### 2.4. Phenotyping of Blast Resistance in the Field

To evaluate the rice blast resistance of the newly improved TGMS lines, the blast resistance experiment was conducted under natural conditions in a rice blast disease hotspot location, Daweishan Village of Liuyang City, Hunan Province, China, where rice blast disease is epidemic every year. One hundred seeds of C5S and every newly improved PTGMS line were sown with the susceptible control CO39, and then CO39 was sown around them. About 30 days after seeding, the disease resistance was visually scored according to a 0–9 SES scale (Standard Evaluation System for Rice, IRRI, 2002) when the susceptible control CO39 showed more than 90% dead seedlings. Twenty random plants in the middle of each line were examined, and the average score was used to measure the disease resistance level of each line. Scores 0–3 were considered as resistant (R), 4 as moderately resistant (MR), 5 as moderately susceptible (MS) and 6–9 as susceptible (S).

### 2.5. Agronomic Performance Evaluation of Rice Blast Resistance Improved Lines and Chuang5S

The MF_3_ plants of newly improved TGMS lines and plants of C5S (CK) were transplanted in the field with a space of 20 cm × 20 cm at the Jiangbei experimental field of Hunan Agricultural University, Hunan Province, in the summer of 2018. The main agronomic characters of five individual plants randomly selected from each line were examined, including plant height, panicle length, flag leaf length, number of primary branches on the panicle, number of grains per panicle and length of panicle exertion. One-way ANOVA was performed to detect the statistical differences. A *p*-value less than 0.05 was considered to be a significant difference.

### 2.6. Yield and Agronomic Performance Evaluation of Hybrid Rice Combinations Developed from the Improved Lines and Chuang5S

Thirty-five hybrid rice combinations crossed between 7 PTGMS lines and 5 restorer lines were evaluated during the summer of 2022. The evaluation of agronomic traits under the natural field condition was conducted in the field at the Jiangbei experimental field of Hunan Agricultural University, Hunan Province. Each combination was planted in a plot of 6 rows with 8 plants per row with a space of 20 cm × 20 cm. At maturity, five plants in the middle of each plot were taken randomly for measurements of the plant height, panicle length, number of panicles per plant, number of grains per panicle, spikelet fertility, 1000 grain weight and yield per plant. One-way ANOVA was performed to detect the statistical differences. A *p*-value less than 0.05 was considered to be a significant difference.

## 3. Results

### 3.1. Development of Gene Pyramided Lines of C5S through MAS

In this study, three backcrossing events were conducted to pyramid the rice blast resistance genes in PTGMS line C5S. At the first step, three rice blast resistance genes (*Pigm*, *Pi48* and *Pi49*) from three donor parents (GM4, XZ3150 and MWG) were introgressed into C5S via marker-assisted backcrossing. F_1_ generation was obtained in the summer of 2011. Then, three successive backcrossing generations were conducted with C5S as the recurrent parent, and BC_3_F_1_ generation was developed in the summer of 2013. For each backcross generation, the presence of disease resistance genes in individual plants was detected using molecular markers; only the individuals harboring the target genes and showing similar morphological phenotypes to their recurrent parent C5S were selected for the following steps. Subsequently, the BC_3_F_1_ and its descendant pedigree individual plants selected via MAS and field phenotyping were handled with continuous irrigation of cold water with a temperature about of 20–22 °C in summer at Changsha, or were planted under low temperature conditions in winter at Sanya to restore the male fertility. Finally, after two times of self-pollination, the BC_3_F_3_ generation was obtained in the spring of 2014.

The second step was to pyramid two blast resistance genes. The hybridization between the BC_3_F_3_ population harboring different rice blast resistance genes of the first step was carried out at Changsha in the summer of 2014. The F_1_ plants were genotyped using molecular markers, and the individuals with heterozygous genotype of target genes were selected for self-pollination; the selected plants were handled with cold water to recover the male fertility in Changsha. The F_2_ populations carrying two rice blast resistance genes were obtained in the summer of 2016.

The third step was to pyramid three blast resistance genes. The F_2_ plants with two rice blast resistance genes were selected for hybridization to obtain multiple F_1_ populations at Changsha in the summer of 2016. In each subsequent generation, the rice blast resistant gene loci were detected using molecular markers, followed by agronomic traits selection in the field; finally, selected individuals were induced to restore fertility to harvest seeds. In the MF_1_ generation, the plants with a heterozygous genotype of blast resistant genes were selected, and in the MF_2_ or descendant generations, the plants with a homozygous genotype of blast resistant genes were selected.

### 3.2. Blast Resistance of the Pyramided Lines

**Figure 2:**
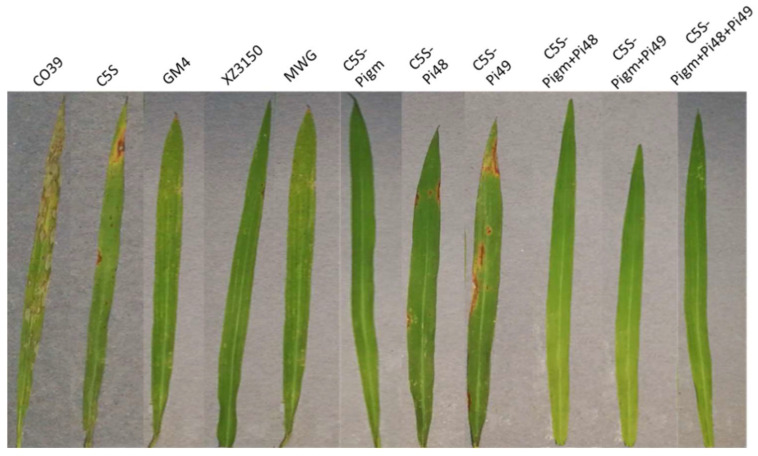
Leaf blast resistance of C5S and the improved lines at seedling stage.

**Figure 3:**
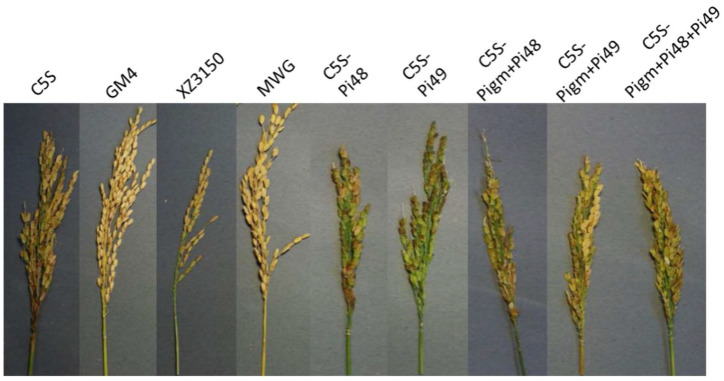
Panicle blast resistance of C5S and the improved lines at grain filling stage.

The pyramided lines carrying homozygous blast resistance genes were selected for the evaluation of leaf blast resistance at the seedling stage and panicle blast resistance at the grain filling stage. The scores of the recurrent parent C5S and the susceptible control CO39 were 6.2 and 9.0, respectively. Three donor parents, GM4, XZ3150 and MWG, were resistant to leaf blast with scores of 1.6, 3.2 and 4.0, respectively. The introgression lines carrying single blast genes *Pigm, Pi48* and *Pi49* showed similar resistance to the donor, with resistance scores of 1.8, 3.6 and 4.8, respectively. In the two-gene pyramided lines, the resistance scores of *Pigm + Pi48* and *Pigm + Pi49* improved lines were 1.6 and 1.8, respectively, which were similar to *Pigm* monogenic lines and *Pigm* donor GM4, but significantly lower than *Pi48* and *Pi49* monogenic lines. The three-gene pyramided lines, including *Pigm*, *Pi49* and *Pi48*, showed comparable resistance to the donor GM4 with resistance grades of 1.6, which were significantly stronger than other monogenic lines and digenic lines (Table 2, Figure 2). At the grain filling stage, the disease reaction scores of all improved materials were 3–5, while the susceptible control CO39 and recurrent parent C5S showed more than 50% death (Table 2, Figure 3). The results revealed that the blast resistance of the newly developed lines was significantly improved in comparison to C5S.

### 3.3. Genetic Background Examination of the Pyramided Lines

**Figure 4:**
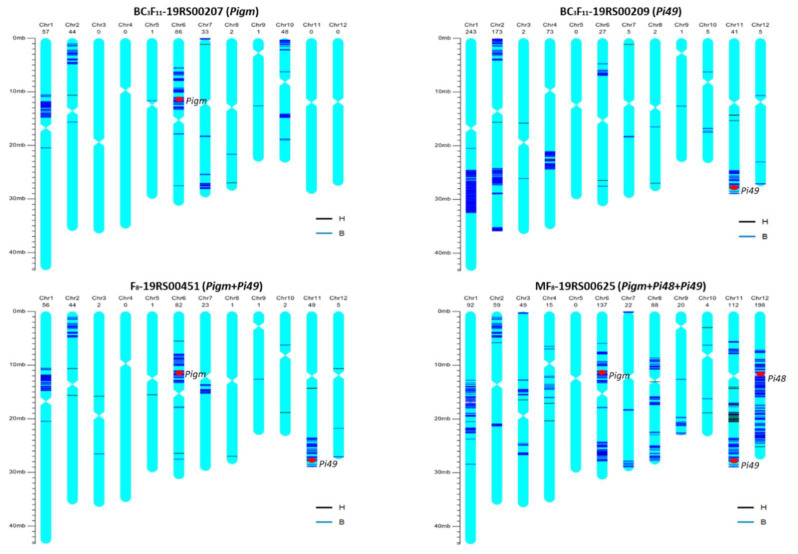
Genetic background of improved lines using the 10K array. The blue color represents homozygous genotype different from C5S, and the black color represents heterozygous genotype. The red dots indicate the positions of the target blast resistance genes.

In this study, a 10K whole-genome SNP array was used to analyze the genetic background of the gene pyramided lines. The genetic background recovery rates of the improved lines 19RS00207 (*Pigm*) and 19RS00209 (*Pi49*) were 97.26% and 94.34%, respectively. The genetic background recovery rate of the digenic line 19RS00451 (*Pigm* + *Pi49*) was 97.53%. The genetic background recovery rate of the trigenic line 19RS00625 (*Pigm + Pi48 + Pi49*) was 92.68%. Fragments carrying target genes were substituted in the positions (red dots) of *Pigm* in chromosome 6, *Pi48* in chromosome 12 and *Pi49* in chromosome 11, indicating the successful introgression of blast resistance genes (Figure 4).

### 3.4. Agronomic Traits of the Pyramided Lines

The main agronomic traits of the pyramided lines and their recurrent parent C5S were evaluated; the results showed that the agronomic traits such as plant height, number of grains per panicle and panicle length of the improved lines were lower than recurrent parent C5S. Among the improved lines, the agronomic traits of C5S-3R-4 were similar to the recurrent parent, and its plant height was not significantly different from C5S. The improved line C5S-3R-3 performed poorly in agronomic traits, and its number of grains per panicle was significantly lower than the recurrent parents (Table 3).

### 3.5. Agronomic Traits of the Hybrid Rice Combinations

In order to study the effect of gene introgression on the agronomic characters of hybrid rice combinations, seven male sterile lines were crossed with five restorer lines, and thirty-five hybrid combinations were obtained for investigation of agronomic traits. The results showed that most of the agronomic traits of the combinations derived from four restorer lines (R1128, HRZ, MFZ1 and YNSM) were similar to those combinations from C5S. In the SFSM group, although panicle length and number of grains per panicle of some combinations from improved lines was significantly higher than the combination from C5S, the 1000-grain weight of these lines were significantly lower than that of the control, and there was no significant difference in the plant height, number of panicles per plant and spikelet fertility, so there was no significant difference in yield (Table 4).

## 4. Discussion

Marker-assisted selection is an effective way to improve the disease resistance of rice; the use of MAS can determine the resistance and fertility of individual plants as early as possible, and the selected individuals are treated with low temperature to restore fertility, thus greatly improving the selection efficiency and shortening the breeding procedure. The blast resistance gene *Pi2* was introgressed into TGMS line C815S through MAS and phenotypic selection approaches, and the agronomic and grain quality traits of four new TGMS lines met the requirement for two-line hybrid rice production [16]. Two PTGMS lines, Tai S and Wo S, were developed using the broad-spectrum resistance gene *Xa23* through MAS combined with phenotypic selection [17]. The blast and bacterial blight resistance of PTGMS line Guangzhan63-4S was improved by introducing the R genes *Pi2* and *Xa7* through MAS [18]. The rice blast and brown planthopper resistance of PTGMS line C815S was improved via the MAS of the *Pi9*, *Pi47*, *Pi48*, *Pi49*, *Bph14* and *Bph15* genes [19]. Similar to previous studies, this study combined a traditional backcross method with MAS to pyramid three blast resistance genes into sterile lines of C5S through several generations of hybridization and backcrossing. The genotyping results indicate that target genes were successfully introgressed into the improved lines. The new developed lines conferred high resistance to rice blast in the field. The genetic background recovery rates were 97.26% and 94.34% for the improved monogenic lines 19RS00207 (*Pigm*) and 19RS00209 (*Pi49*), respectively, and 97.53% for the digenic line 19RS00451 (*Pigm* + *Pi49*) and 92.68% for the trigenic line 19RS00625 (*Pigm + Pi48 + Pi49*). There were more fragments introduced into the recurrent parent when more genes were pyramided, and these fragments may carry genes affecting agronomic traits. Thus, some of the agronomic traits such as the plant height, number of grains per panicle and panicle length of the trigenic lines were lower than recurrent parent C5S (Table 3). However, the effects of the introduced fragments on the performance of the hybrids were dependent on the restore lines. Among the five restore lines used, the 1000-grain weight of hybrids from SFSM were lower than those from the control C5S, while no difference was observed in hybrids from other restore lines (Table 4). Importantly, the hybrid rice combinations of improved lines showed no significant difference in yield compared to the control. The developed PTGMS lines with blast resistance and a similar yield as C5S could be used for the hybrid rice seeds production.

In the practice of crop disease resistance breeding, repeated use of a single gene for a long time can easily lead to the loss of resistance, thus pyramiding broad-spectrum resistance genes is generally considered as an effective way to solve this problem. Multi-gene pyramiding, which is conducive for broadening the resistance spectrum and improving crop resistance, is an important approach for rice variety improvement [20]. Genes *Pi46* and *Pita* were pyramided into an elite restorer line HH179 to improve its blast resistance using the marker-assisted backcrossing procedure, and the resistance spectrum of the three improved lines was markedly broader than that of HH179 [21]. The blast resistances of a japonica rice variety 07GY31 was improved by pyramiding R genes *Pi9*, *Pizt* and *Pi54* [22]. The blast resistances of four sterile lines were improved by the introgression of broad-spectrum blast resistance genes *Pi37*, *Pit*, *Pid3*, *Pigm*, *Pi36*, *Pi5*, *Pi54*, *Pikm* and *Pb1* [23]. The disease resistances of two cultivars (ASD 16 and ADT 43) were enhanced through the introgression of bacterial blight (*xa5*, *xa13*, and *Xa21*), blast (*Pi54*) and sheath blight (*qSBR7-1*, *qSBR11-1* and *qSBR11-2*) resistance genes [24]. In our previous study, we pyramided two rice blast resistance genes, *Pi9* and *Pi49*, into C5S, and the results showed that the resistances of C5S-Pi9/Pi49 lines were improved when different blast strains were inoculated [12]. In this study, we developed lines containing two or three blast resistance genes that were resistant to leaf and panicle blast under natural conditions. The three-gene pyramiding lines of C5S (*Pigm* + *Pi48* + *Pi49*) have higher rice blast resistance levels than the monogenic line and digenic lines. In addition, the present study results also reveal that the gene combination with *Pigm* had better resistance than the remaining improved lines. These results were consistent with the results of previous studies. The improved lines containing one, two or three R genes can be used to breed two-line hybrid rice with broad-spectrum resistance and can also be used to polymerize more resistance genes into intermediate materials via MAS to obtain new materials with a broader resistance spectrum, wider adaptation range and durable resistance. In addition, they can be pyramided with more genes of important agronomic traits, such as the low accumulation of heavy metals and superior grain quality.

In this study, molecular markers linked to the target gene were used for foreground selection, but genome-wide markers were not used for background selection in each generation due to the cost. The genetic background examination results showed that some non-target genomic regions of the higher generation materials still retained genotypes of the donor, especially the large chromosomal segment of *Pi48*. Similar results have been reported in previous studies. A large flanking fragment remained in the recurrent parent when introgressing gene *Pita* to improve rice blast resistance [25]. The *Pi48* gene was the allele of *Pita*, and the *Pita* locus is close to the centromere region, which has a low recombination rate. The background selection is considered to be an effective way to improve the efficiency of recombination selection and reduce the transmission of linkage drag. With the advances in high-throughput genotyping technology and the reduction in genotyping costs, it has become easier to obtain high-quality whole genome genotype. New genotyping technology such as KASP, whole genome sequencing, genotyping via target sequencing and SNP breeding chips have been widely applied in recent years [26,27,28,29,30]. The use of an SNP chip for genetic background selection in each generation can select individuals with higher genomic background recovery as early as possible and shorten the breeding process, and, therefore, it has been widely employed in breeding programs. The RICE6K SNP array was used for background detection in backcross breeding, and obtained improved lines, with a genome recovery of 99.67% [31]. In the case of genomics-assisted breeding, the background analysis using the 56K SNP chip revealed that the selected BC_2_F_3_ line has a background recovery rate of 96.8% comparing to the recurrent parent [32]. The use of the SNP chip for background selection in the process of backcross breeding can reduce the near isogenic line development by 2–3 years, which will further shorten the breeding cycle [31]. Using a liquid chip based on targeted sequencing technology [33] to further improve breeding efficiency is our future research direction on the basis of the improved lines in this study. Using a genomics-assisted breeding strategy to polymerize more important genes and create a series of better C5S is our next research focus.

## Figures and Tables

**Table 1:** Molecular markers used to select individuals with the resistance genes.

Gene	Chr	Marker	Marker Position	Forward Primer	Reverse Primer
*Pigm*	6	RM7311	11046701-11046847	agtggtcgttgaactcggag	tcgtggcgcctttaatctc
		RM7178	10199892-10200042	taaccttcacagcgaacgtg	ccgtgagatgggctacctac
*Pi48*	12	LY2	11935927-11936129	attacgctcgatagtggc	ctagcgggaggttggaag
*Pi49*	11	RM224	27673251-27673353	atcgatcgatcttcacgagg	tgctataaaaggcattcggg

**Table 2:** Blast resistance of C5S and improved lines.

Materials	Blast Resistance Gene	Leaf Blast Resistance at Seedling Stage	Panicle Blast Resistance at Grain Filling Stage
CO39	-	9.0 ± 0 **	9
C5S	-	6.2 ± 0.5	7
GM4	*Pigm*	1.6 ± 0.2 **	3
XZ3150	*Pi48*	3.2 ± 0.4 **	3
MWG	*Pi49*	4.0 ± 0.3 **	5
C5S-Pigm	*Pigm*	1.8 ± 0.4 **	3
C5S-Pi48	*Pi48*	3.6 ± 0.5 **	5
C5S-Pi49	*Pi49*	4.8 ± 0.4 **	5
C5S-Pigm + Pi48	*Pigm + Pi48*	1.6 ± 0.2 **	3
C5S-Pigm + Pi49	*Pigm + Pi49*	1.8 ± 0.4 **	5
C5S-Pigm + Pi48 + Pi49	*Pigm + Pi48 + Pi49*	1.6 ± 0.2 **	3

** Means the disease severity score difference between improved lines and C5S was significant at 0.01 level. For blast resistance, scores 0–3 are resistant, score 4 is moderately resistant, score 5 is moderately susceptible and scores of 7–9 are susceptible.

**Table 3:** Agronomic traits of C5S and the improved lines.

Lines	Genes	Plant Height (cm)	Panicle Length (cm)	Flag-Leaf Length (cm)	Number Primary Branches	Number of Grains per Spike	Length of the Upmost Internode (cm)	Length of Panicle Exertion (cm)
C5S		62.7 ± 0.7	22.1 ± 0.3	34.1 ± 1.4	11.0 ± 0.2	176.7 ± 7.7	20.7 ± 0.4	8.4
C5S-3R-1	*Pigm + Pi48 + Pi49*	54.5 ± 0.8 **	19.9 ± 0.2 **	27.6 ± 1.0 **	9.1 ± 0.3 **	104.8 ± 3.9 **	16.2 ± 0.5 **	10.3
C5S-3R-2	*Pigm + Pi48 + Pi49*	53.9 ± 0.6 **	18.7 ± 0.3 **	25.0 ± 0.6 **	9.8 ± 0.2 **	115.5 ± 4.6 **	16.7 ± 0.4 **	11.7
C5S-3R-3	*Pigm + Pi48 + Pi49*	50.0 ± 0.5 **	17.5 ± 0.2 **	18.9 ± 1.0 **	6.9 ± 0.2 **	77.7 ± 2.8 **	16.8 ± 0.5 **	7.5
C5S-3R-4	*Pigm + Pi48 + Pi49*	62.1 ± 0.8	19.7 ± 0.3 **	27.1 ± 1.0 **	10.0 ± 0.4 **	140.1 ± 6.9 **	21.9 ± 0.4 **	6.7
C5S-3R-5	*Pigm + Pi48 + Pi49*	59.8 ± 0.9 **	18.9 ± 0.3 **	27.2 ± 0.9 **	9.3 ± 0.4 **	112.1 ± 6.1 **	20.6 ± 0.5	6.8

** means there is a significant difference at 0.01 level compared with C5S.

**Table 4:** Agronomic traits of the hybrids of C5S and the improved lines.

Materials	Restorer Lines	PTGMS Lines	Gene	Plant Height (cm)	Panicle Length (cm)	Panicles per Plant	Grain Number per Panicle	Spikelet Fertility (%)	1000-Grain Weight (g)	Yield per Plant (g)
22RC11	R1128	C5S	-	100.7 ± 1.0	20.8 ± 2.4	10.0 ± 1.0	178.7 ± 27.4	78.8 ± 2.9	25.1 ± 0.8	35.5 ± 8.4
22RC12		21YCS01	*Pi49*	115.2 ± 1.9 **	22.6 ± 0.4	9.0 ± 1.0	184.5 ± 10.4	81.6 ± 1.6	24.4 ± 0.2	33.1 ± 5.1
22RC13		21YCS22	*Pi48*	101.7 ± 2.4	23.3 ± 1.7	10.0 ± 3.0	216.8 ± 15.6	87.7 ± 4.6	23.9 ± 2.0	44.9 ± 12.1
22RC14		21YCS23	*Pigm*	106.1 ± 4.6	22.1 ± 1.3	9.7 ± 0.6	209.6 ± 11.8	82.6 ± 3.1	26.1 ± 2.7	43.7 ± 5.1
22RC15		21YCS24	*Pigm + Pi49*	101.9 ± 1.6	22.3 ± 1.9	9.3 ± 0.6	211.7 ± 26.3	79.2 ± 1.0	25.1 ± 1.8	39.2 ± 4.9
22RC16		21YCS61	*Pigm + Pi48*	111.1 ± 2.9 **	21.9 ± 1.0	8.7 ± 0.6	212.4 ± 33.0	83.1 ± 7.0	29.3 ± 1.6 **	45.1 ± 10.5
22RC17		21YCS25	*Pigm + Pi48 + Pi49*	102.3 ± 1.3	21.1 ± 0.8	7.3 ± 0.6	202.4 ± 33.7	88 ± 1.6	26.4 ± 0.5	34.5 ± 6.6
22RC21	HRZ	C5S	-	109.7 ± 2.7	23.1 ± 0.7	11.0 ± 0.0	229.0 ± 28.7	86.7 ± 1.7	24.0 ± 0.2	52.5 ± 7.9
22RC22		21YCS01	*Pi49*	106.0 ± 1.1	20.4 ± 0.8 *	15.3 ± 1.2 **	168.2 ± 18.9 *	89.4 ± 0.7	23.3 ± 0.5	53.4 ± 2.3
22RC23		21YCS22	*Pi48*	101.1 ± 5.3 *	21.7 ± 0.9	11.0 ± 1.0	206.7 ± 9.5	80.1 ± 6.2	22.9 ± 1.0	42.3 ± 10.4
22RC24		21YCS23	*Pigm*	106.7 ± 0.5	22.5 ± 0.9	10.7 ± 0.6	213.0 ± 20.0	85.6 ± 2.5	23.7 ± 0.9	46.3 ± 6.8
22RC25		21YCS24	*Pigm + Pi49*	113.1 ± 2.1	24.7 ± 0.2	9.0 ± 1.0	241.9 ± 14.7	92.0 ± 2.2	22.7 ± 0.5	45.4 ± 3.8
22RC26		21YCS61	*Pigm + Pi48*	108.7 ± 0.6	24.4 ± 0.3	11.3 ± 0.6	243.2 ± 10.8	88.3 ± 1.0	22.6 ± 0.2	55.1 ± 4.4
22RC27		21YCS25	*Pigm + Pi48 + Pi49*	112.4 ± 2.9	23.6 ± 0.8	9.0 ± 1.7	233.9 ± 25.6	87.9 ± 2.9	23.8 ± 0.1	44.2 ± 11.7
22RC31	MFZ1	C5S	-	101.8 ± 1.5	23.2 ± 0.5	13.0 ± 2.0	181.9 ± 11.8	82.8 ± 0.7	22.9 ± 0.8	44.5 ± 4.0
22RC32		21YCS01	*Pi49*	112.1 ± 4.8 *	24.7 ± 0.6	12.7 ± 1.2	209.7 ± 2.9	80.2 ± 1.5 *	23.1 ± 0.9	49.0 ± 2.5
22RC33		21YCS22	*Pi48*	103.5 ± 0.8	22.0 ± 0.9	14.0 ± 1.0	166.4 ± 16.4	86.8 ± 5.6	23.4 ± 0.3	47.2 ± 5.1
22RC34		21YCS23	*Pigm*	104.4 ± 6.2	22.9 ± 0.5	11.7 ± 0.6	208.9 ± 23.1	80.6 ± 5.5	21.5 ± 1.1	42.2 ± 6.3
22RC35		21YCS24	*Pigm + Pi49*	100.8 ± 2.3	23.5 ± 0.3	11.7 ± 0.6	207.8 ± 6.1 *	88.2 ± 1.9	20.8 ± 0.2 **	44.6 ± 3.1
22RC36		21YCS61	*Pigm + Pi48*	97.7 ± 3.9	22.4 ± 0.3	16.0 ± 1.7	191.3 ± 12.4	78.8 ± 1.2	22.8 ± 1.4	54.8 ± 3.3
22RC37		21YCS25	*Pigm + Pi48 + Pi49*	101.4 ± 2.9	23.0 ± 0.9	12.7 ± 0.6	199.0 ± 13.4	85.3 ± 4.0	22.9 ± 0.3	49.3 ± 5.3
22RC41	SFSM	C5S	-	111.9 ± 5.0	20.9 ± 0.7	15.0 ± 1.0	151.3 ± 19.9	87.9 ± 2.9	24.4 ± 0.9	55.6 ± 6.2
22RC42		21YCS01	*Pi49*	103.2 ± 2.6	21.5 ± 1.0	14.0 ± 1.0	165.0 ± 3.8	81.8 ± 1.2	22.8 ± 0.4 *	43.1 ± 4.3
22RC43		21YCS22	*Pi48*	110.3 ± 3.6	23.9 ± 0.4 **	12.3 ± 1.5	227.0 ± 6.1 **	79.8 ± 7.7	21.3 ± 0.4 **	47.4 ± 4.7
22RC44		21YCS23	*Pigm*	119.3 ± 0.8	23.2 ± 0.4 *	13.0 ± 1.0	220.4 ± 13.7 **	87.0 ± 0.8	22.5 ± 0.4 *	56.0 ± 2.6
22RC45		21YCS24	*Pigm + Pi49*	121.0 ± 1.0	24.0 ± 0.2 **	12.3 ± 0.6	208.0 ± 2.6 **	81.0 ± 0.2	22.7 ± 0.2 *	47.1 ± 2.5
22RC46		21YCS61	*Pigm + Pi48*	99.9 ± 7.5 *	23.4 ± 1.3 *	13.0 ± 1.7	214.5 ± 9.2 **	86.5 ± 3.3	21.7 ± 1.0 *	52.2 ± 4.6
22RC47		21YCS25	*Pigm + Pi48 + Pi49*	117.5 ± 1.6	24.2 ± 0.1 **	11.3 ± 1.2 *	238.5 ± 17.1 **	88.6 ± 3.7	22.1 ± 0.2 **	52.7 ± 5.3
22RC51	YNSM	C5S	-	104.2 ± 6.2	22.8 ± 0.7	15.7 ± 1.5	177.9 ± 22.4	79.8 ± 0.9	23.8 ± 0.2	52.6 ± 1.7
22RC52		21YCS01	*Pi49*	105.3 ± 3.3	24.3 ± 1.2	12.0 ± 1.0 *	263.3 ± 30.2 **	76.8 ± 4.6	23.4 ± 1.5	56.6 ± 5.8
22RC53		21YCS22	*Pi48*	106.0 ± 0.4	23.7 ± 0.8	11.7 ± 0.6 **	241.7 ± 25.6	78.2 ± 5.0	23.4 ± 0.2	51.7 ± 8.9
22RC54		21YCS23	*Pigm*	106.6 ± 1.4	24.6 ± 0.9	11.3 ± 0.6 **	230.7 ± 9.2	79.3 ± 5.0	24.5 ± 0.9	50.7 ± 1.7
22RC55		21YCS24	*Pigm + Pi49*	104.0 ± 1.6	23.6 ± 0.8	13.0 ± 1.0 *	226.3 ± 20.8	84.8 ± 1.8	22.3 ± 1.0	55.8 ± 9.6
22RC56		21YCS61	*Pigm + Pi48*	104.6 ± 3.5	23.0 ± 1.4	11.0 ± 0.0 **	241.9 ± 25.8	82.3 ± 7.3	22.7 ± 0.1	49.9 ± 9.0
22RC57		21YCS25	*Pigm + Pi48 + Pi49*	102.4 ± 2.2	22.7 ± 0.2	13.7 ± 0.6	212.8 ± 21.9	85.9 ± 3.4	22.8 ± 0.4	56.8 ± 5.9

* and ** means there is a significant difference at 0.05 and 0.01 level compared with hybrids from C5S.

## Data Availability

Not applicable.
